# Ovarian reaction and estrus manifestation in delayed puberty gilts after treatment with equine chorionic gonadotropin

**DOI:** 10.1186/1477-7827-10-61

**Published:** 2012-08-22

**Authors:** Ivan B Stančić, Darko V Bošnjak, Ivan B Radović, Blagoje L Stančić, Roger B Harvey, Robin C Anderson

**Affiliations:** 1 University of Novi Sad, Faculty of Agriculture, 21000 Novi Sad, Trg D. Obradovića 8, 21000, Novi Sad, Serbia; 2 Food and Feed Safety Research Unit, ARS, U.S. Department of Agriculture, College Station, TX, 77845, USA

**Keywords:** Delayed puberty, Ovarian reaction, Estrus, eCG, Gilts

## Abstract

**Background:**

Prolonged pre-insemination anestrus (i.e. delayed puberty) is a major contributing factor for culling up to 30% of the replacement gilts at large breeding farm units in Vojvodina. It is imperative to determine if these gilts are acyclic (prepubertal) or cyclic, but just fail to exhibit behavioural estrus. Recent investigations demonstrate that treatment with equine chorionic gonadotropin (eCG) can increase the diestrous phase duration in sexually mature gilts. Based on these finding, the aim of the present studies was to determine the reproductive status of delayed puberty gilts following injection with eCG.

**Methods:**

Two experiments were conducted on a swine breeding farm in Vojvodina. In Exp. 1, 20 prepubertal (acyclic) gilts, and 120 sexually mature (cyclic) gilts were injected with a single injection of 400 IU eCG + 200 IU human chorionic gonadotropin (hCG) or with 1000 IU eCG (cyclic gilts), at d5, d11 or d17 after spontaneous estrus detection, to determine their ovarian reaction and induced estrus manifestation. In Exp. 2, sixty delayed puberty gilts (estrus not detected until 8 month of age, av. 258 days) were culled from breeding herd and slaughtered to determine their reproductive status based on ovarian anatomical features. The second group of gilts (n = 60) was treated with a single 1000 IU eCG injection to determine their reproductive status, based on the interval between eCG injection to estrus detection and duration. The data were analyzed by descriptive statistics, *t*-test, analysis of variance and Duncan’s test in the software package Statistics 10^th^.

**Results:**

Ovulations were induced in 90% of acyclic (sexually immature) and, on average, 93.3% of cyclic (sexually mature) gilts after the eCG injection. On average, 4 days after the eCG injection, estrus was detected in 85% of the treated acyclic (sexually immature) gilts and in 95% (19/20) of the cyclic (sexually mature) gilts, treated with eCG on day 17 after spontaneous estrus detection. The interval from eCG to induced estrus detection was prolonged (av. 25 days) in 95% (19/20) of the sexually mature gilts treated with eCG on day 5 and in 90% (18/20) of gilts treated on day 11 after spontaneous estrus detection (Exp. 1). Forty anestrous gilts reached cyclic pubertal ovarian activity. Estrus manifestation was detected in 56 gilts (93.3% of the total 60 treated prolonged anestrous gilts, av. 259 days of age), after a single 1000 IU eCG injection. Thirty-four gilts (60.7% of the total gilts in estrus) with prolonged eCG to estrus interval (av. 24.7 days) were considered spontaneously cyclic (sexually mature), but behaviourally anestrous before treatment. The remaining 22 (39.3% of the total gilts in estrus) were considered truly sexually immature (acyclic) before the treatment or were eCG injected in the late luteal or proestrous phase of spontaneous estrous cycle (Exp. 2).

**Conclusions:**

In 66.7% of the delayed puberty gilts, pre-ovulatory follicles (PoF), corpora hemorrhagica (CH), corpora lutea (CL), or corpora albicantia (CA) were found on the ovaries upon post mortem examination. These gilts were considered as sexually mature before slaughtering. In 60.7% of the delayed puberty gilts, behavioural estrus was detected an average of 24.7 days following eCG injections. These gilts were considered as eCG treated during the luteal phase (diestrus) of the spontaneous estrus cycle. Both findings suggest that delayed puberty gilts actually reached cyclic pubertal ovarian activity (sexual maturity) before culling from the breeding herd.

## Background

In order to achieve successful weaned swine production, sufficient service-ready gilts must be available in each breeding week for efficient use of gestation and farrowing facilities, and as replacements for sows culled from the breeding herd [1]. However, this goal is, in practice, quite difficult to achieve, because more gilts are culled from the herd before the first insemination [2]. Recent investigation on large swine breeding farms in Vojvodina demonstrated that 30 to 40% of gilts failed to exhibit behavioural estrus even after 8 months of age. These gilts are culled from the breeding herd as delayed puberty gilts, i.e. prolonged pre-insemination anestrous gilts [3,4]. Truly delayed anestrus can be the result of sterol dysregulation, thereby disrupting oocyte meiosis and primordial follicle formation [5,6]. However, behavioural delayed anestrus may be the result of poor estrus detection by farm personnel [7].

Prolonged prepubertal anestrus, i.e. delayed puberty (when estrus is not detected in gilts older than 8 months of age) is the most common reason for culling gilts from the breeding herds [8,9]. However, post mortem examination of reproductive organs has shown that many delayed puberty gilts actually have cyclic ovarian activity [10-13]. Based on these finding, it can be concluded that such gilts are not truly prepubertal anestrous. It is important to determine whether delayed puberty gilts are truly acyclic (prepubertal) or cyclic but fail to exhibit behavioural estrus before their culling from the breeding herd. Treatment with gonadotropins can be a method for reproductive status determination. Namely, delayed puberty gilts that are truly acyclic will respond to exogenous gonadotropins with estrus and ovulation rates similar to those gilts in which puberty is induced at a much younger age. However, if behaviourally anestrous gilts are treated during the luteal phase, diestrus will be prolonged [11,14,15].

The aim of this work was to determine ovarian reaction and estrus manifestation in delayed puberty gilts after treatment with single eCG injection.

## Methods

The experiment was conduced at one commercial farm unit from March to May, 2011 in terms of regular production cycle. The moust of the our research are based on regular terms of production cycles in farms, with no specific exposure to animal suffering.

### First experiment

This study was conducted to determine ovarian reaction and estrus manifestation in prepubertal (acyclic) gilts, about 160 ± 5 days of age (n = 20), and sexually mature (cyclic) gilts with at least one spontaneous estrus cycle, about 210 ± 5 days of age (n = 120), after treatment with gonadotropin preparations. Prepubertal (sexually immature, acyclic) gilts (n = 20) were treated with a single intramuscular injection 400 IU eCG + 200 IU hCG (PG600®, Intervet International B.V., Boxmeer, The Netherlands). Estrus detection was performed twice within 24 h (at an interval of about 12 h) by direct boar contact. Gilts were sent to slaughter according to the regular schedule in a registered slaughterhouse 14 days after eCG injection for a post mortem ovarian examination. Sexually mature (cyclic) gilts were treated with a single intramuscular injection 1,000 IU eCG (“Sugonal”, Veterinary Institute Subotica), at 5 (n = 40), 11 (n = 40) or 17 days (n = 40) after spontaneous estrus manifestation (Day 1 - the onset of estrus). One half of the gilts from each subgroup were sent to slaughter 14 days after eCG injection for a post mortem ovarian examination. The other half of the gilts was tested for estrus manifestation after eCG injection.

### Second experiment

A total of 120 gilts culled from a breeding herd due to prolonged anestrus (estrus not detected until 8 month of age, av. 258 days) were used to: (a) determine the physiological reproductive status (without eCG treatment) based on the post mortem ovarian examination (n = 60) or (b) determine the behavioural estrus manifestation in such gilts, after treatment with single intramuscular injection 1000 IU eCG (n = 60). Not treated gilts (n = 60) were sent to a commercial abattoir for post mortem ovarian examination. Estrus detection in eCG treated gilts was performed twice within 24 h (at an interval of about 12 h) by full boar contact, starting 24 hours after eCG injection.

### Post mortem ovarian examination

Ovaries were examined for the presence of the cyclic functional ovarian structures: PoF (8–10 mm diameter), CH, CL, and CA. Based on the present ovarian structures, the gilts were classified as sexually mature (cyclic) or sexually immature i.e., pre-pubertal (acyclic). Sexually mature gilts were those with ovaries having: (a) PoF, CH, CL, and CA. Gilts were considered sexually immature (acyclic, prepubertal) when their ovaries had follicles ≤ 7 mm in diameter and lacked any other ovarian structures.

### Behavioural estrus manifestation

Gilts in which estrus was detected within the first 7 days after eCG injection were considered as sexually immature (acyclic), or truly acyclic delayed puberty gilts before eCG treatment. Gilts with a prolonged estrous cycle were considered as having been injected with eCG during the luteal phase of the spontaneous estrous cycle (prolonged diestrus). In the group of delayed puberty gilts, such gilts were considered behaviourally anestrous cyclic gilts.

### Analysis of data

Descriptive statistics, *t*-test, analysis of variance (ANOVA) and Duncan’s test were done in the software package Statistics 10^th^. Descriptive statistics was performed on characteristics CL, CA, eCG to estrus (days) and spontaneous-estrus to estrus after eCG (days). In all cases, a Duncan’s test was used to determine differences between means. Changes in CL number, number of CA, eCG-estrus (days) and spontaneous-estrus to estrus after eCG (days) compared to spontaneous-estrus detection to eCG injection (days) in sexually mature gilts were compared by ANOVA and Duncan’s test. Differences in the number of CL and CA compared to spontaneous-estrus detection to eCG injection (days) were made by a *t*-test. The difference between sexually mature (cyclic) and sexually immature (acyclic) gilts and the number of CL were tested by ANOVA and Duncan’s test. The statistical significance was set at P < 0.05.

## Results

The percentage of gilts that ovulated after eCG injection was similar for sexually mature gilts treated on day 5 (95%), 11 (90%) or 17 (95%) when compared to sexually immature, prepubertal gilts (90%). The average ovulation rate in sexually mature gilts was 18.95 CL and not significantly different (p > 0.05) between treatment groups. The average ovulation rate in treated sexually immature gilts (11.78 CL) was significantly smaller (p < 0.05) compared to sexually mature gilts. The induced average ovulation rate (18.95 CL) was significantly higher (p < 0.05) compared with the average ovulation rate (10.82 CA) in the spontaneous estrous cycle of sexually mature gilts (Table 1). Behavioural estrus was detected in 90% to 95% (av. 93.3%) of treated sexually mature, and in 90% of the treated sexually immature gilts. The intervals between eCG injection and estrus detection, in sexually mature gilts treated on day 5 and day 11 of spontaneous estrous cycle (24.9d and 25.1d), were not significantly different (p > 0.05). However, this interval in gilts treated on day 17 (4.2d) was significantly shorter (p < 0.05), and they were identical (4.1d) with intervals in treated sexually immature gilts. The intervals between spontaneous-estrus and estrus detected after eCG injection were prolonged in sexually mature gilts treated on days 5 and day 11 (33.9d and 40.1d). However, the interval in gilts treated on day 17 were of normal physiological duration (21.2 days) and significantly shorter (p < 0.05) than in gilts treated on d5 and d11 (Table 1).

**Table 1:** Ovarian reaction and estrus manifestation in sexually mature and pre-pubertal gilts treated with eCG (average ± SD)

**Parameter**	**Gilts reproductive status**				
	**Sexually mature (cyclic)**^**†**^			**Total**	**Sexually immature (acyclic)**^**††**^
Spontaneous estrus detection to eCG injection (days)	5	11	17	(5, 11, 17)	-
Gilts with ovulation response after eCG	95% (19/20)	90% (18/20)	95% (19/20)	93.3% (56/60)	90% (18/20)
Average CL^1^ number	18.05 ± 2.48 (12–22)	19.28 ± 4.82 (14–32)	19.63 ± 4.86 (13–34)	18.95 ± 4.17^A^ (12–34)	11.78 ± 3.51^B^ (4–20)
Average CA^2^ number	10.75 ± 2.31 (6–14)	10.45 ± 2.57 (7–15)	11.25 ± 2.30 (7–16)	10.82 ± 2.3^B^ (6–16)	-
Gilts with induced estrus detected	95% (19/20)	90% (18/20)	95% (19/20)	93.3% (56/60)	85% (17/20)
eCG to estrus (days)	24.9 ± 0.62^A^ (24–26)	25.1 ± 0.34^A^ (24–26)	4.2 ± 0.73^B^ (3–6)	-	4.1 (3–5)
Spontaneous estrus to estrus after eCG (days)	33.9 ± 0.61^A^ (33–35)	40.1 ± 0.69^A^ (38–42)	21.2 ± 0.73^B^ (20–23)	-	-

^†^Single i/m injection 1.000 IU eCG. ^††^400 IU eCG + 200 IU hCG 72 h later.

^1^Corpora lutea.

^2^Corpora albicantia per gilt.

^AB^Values with different superscript are statistically significant p < 0.05.

Forty gilts (66.7% of the gilts studied) reached cyclic pubertal ovarian activity before slaughter, having one of the following cyclic ovarian structures: PoF, CH, CL, or CA. The remaining 20 (33.3%) gilts without cyclic ovarian structures were considered as truly acyclic (sexually immature) delayed puberty gilts. The average ovulation rate was 10.7 CH, CL or CA, and the potential ovulation rate was 11.2 (CH, CL or CA + average number of preovulatory follicles, ≥ 8 mm in diameter) (Table 2). Behavioural estrus was detected in 56 delayed puberty gilts (93.3% of the total number of studied gilts), an average of 16.7 days after eCG injection. The average duration of interval from eCG injection to estrus detection was 24.7 days in 60.7%, and 4.3 days in 39.3% from the total of 56 gilts with estrus manifestation. Gilts with a prolonged interval of eCG to estrus (24.7d) were considered sexually mature before treatment. Gilts with 4.3 days eCG to estrus intervals were considered sexually immature (acyclic) before treatment or they were injected with eCG during the late diestrus or proestrus phase of spontaneous estrous cycle (Table 3).

**Table 2:** Post mortem ovarian examination in delayed puberty gilts (n = 60)

**Parameter**	**Value**
Average age at slaughter (days)	257.7 (250 – 268)
Sexually mature (cyclic) gilts^1^	66.7% (40/60)
Sexually immature (acyclic) gilts^2^	33.3% (20/60)
Average ovulation rate^3^	10.7 (6–14)
Average potential ovulation rate^4^	11.2 (5–18)

^1^Functional structures (PoF, CH, CL or CA) present on ovaries.

^2^Ovaries had follicles ≤ 7 mm in diameter and lacked any other ovarian structures.

^3^Average number of CH, CL or CA, present on both ovaries.

^4^Average ovulation rate (CH, CL or CA) + average number of preovulatory follicles (≥ 8 mm in diameter).

**Table 3:** Behavioural estrus manifestation in delayed puberty gilts treated with eCG (n = 60)

**Parameter**		**Value**
Average age at eCG injection (days)		258.7 (248 – 271)
Gilts with behavioural estrus manifestation after eCG		93.3% (56/60)
Average interval eCG to estrus, (days)		16.66 (3 – 27)
eCG – estrus interval distribution, days	24.7 (23 – 27)	60.7% (34/56)
	4.3 (3 – 6)	39.3% (22/56)

## Discussion

In the present study, eCG injections induced estrus and ovulation in the majority of sexually mature and prepubertal gilts. Our results are in agreement with other studies in that a combined injection of eCG and hCG could induce estrus and ovulation in 80 to 90% of prepubertal gilts within 3 to 7 days [16-19]. Furthermore, it is known that an injection of eCG can induce accessory corpora lutea at any stage of the estrous cycle [20-22]. When sexually mature gilts were injected with eCG during the period of functional activity of spontaneous CL (up to day 16 of spontaneous estrous cycle), interestrous intervals were prolonged [18]. It is reported that eCG treatments induce accessory corpora lutea and high level of serum progesterone, thus suppressing behavioural estrus manifestation [11]. However, eCG injections administered during the late luteal phase of the spontaneous estrous cycle are at a time when CL induce a rapid decrease in progesterone secretion. Consequently, induced ovulations are accompanied by behavioural estrus manifestation. Induced ovulation rates were dependent on eCG dose, age, genetic composition and reproductive status of treated gilts [18,19,23-25].

In our experience, approximately 30% of gilts selected for reproduction are culled from the breeding herd because external signs of estrus were not detected even after 8 months of age [3,4,7]. Based on post mortem examination of the reproductive organs of those prolonged anestrous gilts, it was determined that 61% of culled gilts had reached sexual maturity. Furthermore, there were no pathomorphological structures on the reproductive organs of sexually mature culled gilts which could cause anoestrus [14,26].

Functional structures such as PoF, CH, CL, and CA present on the ovaries, would indicate that a gilt is sexually mature and cyclic, whereas a gilt would be acyclic if ovarian follicles were ≤ 7 mm in diameter [12,13,26,27].

Gilts with prolonged intervals from eCG injection to estrus detection were considered spontaneous cyclic (sexually mature), but behaviourally anestrous before treatment. Gilts with normal (4 to 5 days) intervals from eCG injection to estrus detection, considered as truly sexually immature (acyclic) before treatment or were eCG injected in the late luteal or proestrus phase of spontaneous estrus cycle. This conclusion is based on the findings in the present study and those of others [11,14,15,18,20,21,26] that: (1) eCG injection in the luteal phase (up to day 16) of the spontaneous estrous cycle, prolongs interestrous intervals as a result of accessory corpora lutea activity, and (2) induced estrus occurs within 4 to 7 days after eCG injection in sexually immature (acyclic) gilts, as well as in the late diestrus or proestrus of spontaneous sexually mature (cyclic) gilts.

Induction of LH receptor [28], activation of sterol pathways [29,30] and stimulation of epidermal growth factor receptor [31] is the main physiological mechanisms by which eCG treatment induces ovulation, accessory corpora lutea, and high level of serum progesterone.

## Conclusions

The results obtained in the present study indicate that treatment with a single eCG injection can be used as a practical method for determination of the reproductive status (cyclic or acyclic) in prolonged anestrous gilts, before their culling from the breeding herd.

## Abbreviations

CA, Corpus albicans; CH, Corpus haemorrhagicum; CL, Corpus luteum; eCG, Equine chorionic gonadotropin; hCG, Human chorionic gonadotropin; IU, International unit; LH, Luteinizing hormone; PoF, Preovulatory follicle.

## Competing interests

The authors declare that they have no competing interests.

## Authors’ contributions

IBS as the mentor of the doctoral thesis of DVB designed the experiment, contributed in carrying out the experiment, with the analysis of the data and revising critically the contents of the manuscript. DVB contributed in carrying out the experiment, gathered and analyzed the data, and drafted the manuscript. IBR contributed in carrying out the experiment and with contents of the manuscript. BLS contributed in experiment design, data revising, analyzing and discussing. RBH and RCA contributed in the data analysis and critical revision of the manuscript contents. All authors read and approved the final manuscript.
